# DLAT activates EMT to promote HCC metastasis by regulating GLUT1-mediated aerobic glycolysis

**DOI:** 10.1186/s10020-025-01125-5

**Published:** 2025-02-20

**Authors:** Qian Yin, Yinye Yao, Jiaojiao Ni, Yiwen Zhang, Jia Wu, Hui Zeng, Wei Wu, Wei Zhuo, Jieer Ying, Jingjing Li

**Affiliations:** 1 Postgraduate training base Alliance of Wenzhou Medical University (Zhejiang Cancer Hospital), Hangzhou, 310022 Zhejiang China; 2 Department of Hepato-Pancreato-Biliary & Gastric Medical Oncology, Hangzhou Institute of Medicine (HIM), Zhejiang Cancer Hospital, Chinese Academy of Sciences, Hangzhou, 310022 Zhejiang P. R. China; 3 Zhejiang Provincial Clinical Research Center for Malignant Tumor, Hangzhou, 310014 Zhejiang P. R. China; 4 Hepatobiliary and Pancreatic Surgery Department, Hangzhou Institute of Medicine (HIM), Zhejiang Cancer Hospital, Chinese Academy of Sciences, Hangzhou, Zhejiang P. R. China; 5 Department of Interventional Radiology, Hangzhou Institute of Medicine (HIM), Zhejiang Cancer Hospital, Chinese Academy of Sciences, Hangzhou, 310022 Zhejiang P. R. China; 6 Department of Pathology, Hangzhou Institute of Medicine (HIM), Zhejiang Cancer Hospital, Chinese Academy of Sciences, Hangzhou, Zhejiang P. R. China; 7 Department of Cell Biology, Department of Colorectal Surgery and Oncology, Center for Medical Research and Innovation in Digestive System Tumors, The Second Affiliated Hospital, Cancer Center, Ministry of Education, Zhejiang University School of Medicine, Zhejiang University, Hangzhou, China

**Keywords:** DLAT, EMT, GLUT1, Glycolysis, Metastasis, HCC

## Abstract

**Background:**

Metabolic reprogramming is a hallmark of hepatocellular carcinoma (HCC) progression, driving aberrant cellular processes in response to pathological stimuli. While dihydrolipoyl transacetylase (DLAT) has been implicated in the development of various cancers, its specific role and underlying mechanisms in HCC remain unclear. This study aimed to investigate the expression, function, and mechanistic impact of DLAT in HCC.

**Methods:**

A comprehensive analysis was conducted using RNA sequencing data, tissue microarrays, in vitro and in vivo functional assays, and mechanistic studies to evaluate DLAT expression, its functional role in tumor progression, and associated molecular pathways in HCC.

**Results:**

Our study revealed a significant upregulation of DLAT expression in HCC, which was linked to a poor prognosis. Furthermore, we discovered that DLAT facilitated tumor metastasis by driving metabolic reprogramming in HCC cells. Mechanistically, DLAT was found to enhance glucose transporter 1 (GLUT1) expression via H3K18 acetylation, thereby promoting aerobic glycolysis and epithelial-to-mesenchymal transition (EMT), which subsequently augmented metastasis of HCC both in vitro and in vivo. Finally, we confirmed a positive correlation between DLAT and GLUT1 expression in HCC tissues.

**Conclusions:**

These findings establish DLAT as a key regulator in HCC progression and suggest its potential as a promising predictive biomarker and therapeutic target for improving HCC diagnosis and treatment.

**Supplementary Information:**

The online version contains supplementary material available at 10.1186/s10020-025-01125-5.

## Background

Liver cancer, with approximately 906,000 new cases and 830,000 deaths, is the sixth most common cancer and the third leading cause of cancer-related deaths globally (Ladd et al. 2024). Hepatocellular carcinoma (HCC), the predominant form of primary liver cancer, accounts for more than 90% of all liver cancer cases (2012) 2012). HCC has a poor prognosis, with 70–80% of HCC patients having advanced distant metastases at the time of initial diagnosis and being unable to undergo radical surgical resection. Although emerging targeted therapies and immunotherapies have played an important role in the treatment of metastatic HCC, the five-year survival rate is still only 18% (Yang et al. 2019, 2023a, ). Hence, Exploring and understanding the underlying molecular mechanisms of metastatic HCC progression, which are warranted for identifying and validating new potential diagnostic biomarkers and therapeutic targets, is still urgently needed.

Metabolic reprogramming contributes to the development of HCC by inducing aberrant processes in cells in response to pathological signals. Aerobic glycolysis, a typical metabolic reprogramming characterized by the tendency of HCC cells to metabolize glucose into ATP and lactic acid even under well-oxygenated conditions, can modulate the process of proliferation, migration, invasion, and drug resistance of HCC cells (Feng et al. 2020; Guo et al. 2023; Cui et al. 2024). Moreover, lactic acid, once considered a metabolic waste product of glycolysis, has been shown to promote HCC cell proliferation and invasion procession (Yang et al. b ). Therefore, targeting alterations in glycolysis-related tumor metabolism will lay the basis for inhibiting HCC development and reversing HCC therapeutic resistance (Wang et al. 2023; Du et al. 2022).

Dihydrolipoyl transacetylase (DLAT), the structural core E2 component of the pyruvate dehydrogenase complex (PDC), is a multidomain protein(Škerlová et al. 2021). DLAT fuels the classical tricarboxylic acid cycle (TCA) pathway by transferring the acetyl group formed through the oxidative decarboxylation of pyruvate to coenzyme A to generate acetyl coenzyme A (CoA), thus serving as a key hub linking glycolysis and the TCA cycle (Arnold et al. 2022; Stacpoole and McCall 2023). Disruption of interactions between DLAT and the pyruvate dehydrogenase complex component X (PDHX) hinders the assembly of PDC, which can be inactivated, thus promoting a shift from the TCA cycle to aerobic glycolysis and an increase in lactate production, and ultimately promoting tumor progression (Jiang et al. 2024). Previous studies have shown that DLAT plays a crucial role in normal physiological functions and disease progression, such as embryonic development, primary biliary cholangitis, and cellular homeostasis (Terziroli Beretta-Piccoli et al. 2018; Tsvetkov et al. 2022). Although studies have reported that DLAT was associated with poor prognosis in HCC (Ke et al. 2023; Zhou et al. 2022), the underlying molecular mechanisms by which it regulates HCC progression through metabolic reprogramming remain obscure in HCC.

Our study aimed to explore the detailed molecular mechanisms by which DLAT affects the phenotype of HCC via vitro and vivo experiments. Our results demonstrated the role and molecular mechanisms of DLAT in glycose metabolism and development of HCC, which may provide a promising therapeutic target for the treatment of HCC.

## Methods

### Bioinformatics analysis

RNA-sequencing data of 374 patients with HCC along with corresponding clinical information, were obtained from The Cancer Genome Atlas (TCGA) database (https://portal.gdc.cancer.gov). Expression data were transformed to the log_2_(x + 0.001). Prognostic information was obtained from previously published study(Liu et al. 2018). Differences in DLAT expression between HCC and adjacent normal tissues, as well as its association with the pTNM stage, were identified using the “ggplot2” package in R (4.2.1). Kaplan–Meier survival analysis and Cox regression modeling were performed using the “survival” package in R, and results were visualized with the “survminer” package and “ggplot2” package.

### Tumor tissue microarray and immunohistochemistry (IHC)

Tissue microarrays of human HCC samples (*n* = 80) were obtained from Shanghai SuperBiotek Co. Ltd. (Shanghai, China). Tissue microarrays require dewaxing and rehydration for immunohistochemical staining. Protein expression was examined using anti-DLAT or anti-GLUT1 antibodies at 4 °C overnight following by incubating with secondary antibodies. The tissue microarrays were subsequently probed with the streptavidin-alkaline phosphatase system and scanned with 3DHISTECH (Budapest, Hungary, PANNORAMIC Desk/MIDI/250/1000). IHC scores were quantitatively calculated based on staining intensity scores and percentage of stained cells, which were assessed by specialized pathologists. Based on the optimal cut-off value determined by receiver operating characteristic (ROC)curves, the cohort was divided into low and high expression levels of DLAT and GLUT1.

### Cell culture and reagents

Human HCC cell lines Huh7, MHCC-97 H, and PLC/PRF/5 were purchased from Banma Biotechnology (Changsha, China). MHCC-97 L and Hep3B were obtained from Servicebio (Wuhan, China) All cell lines were cultured in DMEM (Servicebio, Wuhan, China) supplemented with 10% fetal bovine serum (Thermo Fisher Scientific, USA) and 1% penicillin/streptomycin (FDbio, China) at 37 °C in a 5% CO_2_ atmosphere. All cell lines were confirmed through short tandem repeat (STR) profiling, and mycoplasma detection was performed before all experiments.

### Cell transfection and infection

According to the manufacturer’s instructions, transient cell transfections of siRNA or plasmid were transfected with Lipo3000 (Invitrogen, Cat No. L3000015). The siRNA oligonucleotides targeting GLUT1 and control siRNA were obtained from RiboBio (Guangzhou, China).

Cell lines stably knocked-down DLAT (sh-DLAT) or overexpressed DLAT(Lv-DLAT) were established using lentivirus vectors (GeneChem Bio-Medical Biotechnology, Shanghai, China), and the transfected cells were selected in puromycin (2 ug/mL) for 1 week. The target sequences of sh-RNAs and si-RNAs are listed in Supplementary Table S1.

### Cell proliferation and colony formation assays

For the CCK-8 assay, 3000–5000 cells were plated into 96-well plates and incubated for 24 h. 10% CCK-8 (TargetMol, C0005, USA) was added to each well for 2–3 h at the indicated time points (0, 12, 24, 48, and 72 h) and measured the absorbance of every well at 450 nm. For colony formation assays, Huh7 and 97 L cells were seeded in 6-well culture plates at a density of 5 × 10^3^ cells/well and cultured with complete mediums for 7–14 days. Visible cell colonies were fixed with 4% paraformaldehyde and stained with 0.1% crystal violet for 30 min at room temperature.

### Wound-healing and transwell assays

HCC cells were plated into 6-well plates to perform the wound-healing assay. We use sterile pipette tip to scratch the cells and take images at 0,24,48, and 72 h after wounding. For Transwell assays, 5–10 × 10^4^ HCC cells were inoculated in the upper transwell chamber(Corning, 3422, USA)with 200 µL serum-free medium, while 800 µL medium supplemented with 10% FBS were added to the bottom chamber. Migrated HCC cells in the lower surface were fixed and stained.

### Lactate, pyruvate, ATP, and glucose uptake assays

Intracellular L-lactate and pyruvate levels in HCC cells were quantified using L-lactate assay kit (A019–2 − 1, Jiancheng Bioengineering Institute, Nanjing, China) and pyruvate assay kit (A081–1–1, Jiancheng Bioengineering Institute, Nanjing, China) according to the manufacturer’s instruction. Absorbance of samples was detected at 530 nm for L-lactate and 505 nm for pyruvate. Intracellular ATP synthesis was detected using an ATP assay kit (S0026, Beyotime, Shanghai, China) according to the manufacturer’s instruction. ATP levels were measured using a microplate reader (Thermo Fisher Scientific, USA). Glucose uptake was detected in HCC cells using a glucose Uptake-Glo™ Assay Kit (Promega, Madison, WI, USA). All in vitro experiments were performed in triplicate.

### Seahorse experiments

HCC cells were seeded into XF24 cell culture microplates (Seahorse, Cat No. 102342-100) at a density of 3 × 10^4^ per well and incubated overnight. At the same time, HCC cells of the same density as the experiment were seeded into per well of a normal 96-well plate for normalization of data analysis. The extracellular acidification rate (ECAR) and oxygen consumption rate (OCR) were measured using the Glycolysis Stress Test Kit (Seahorse, Cat No. 103020-100) and Cell Mito Stress Test Kit (Cat No. 103015-100) according to the manufacturer’s instructions. Normalization of the hippocampus-based glycolysis assay in the data was achieved using CCK-8 detection of the OD value of each well in a control 96-well plate. Three independent replicates were analyzed.

### Western blot and analysis

Tissue and total cell proteins were lysed in RIPA buffer (Beyotime Biotechnology, Jiangsu, China) supplemented with 1% PMSF (FDbio, Hangzhou, China) for 30 min on ice and were subsequently separated on 12.5% SDS-PAGE gels. A TBS solution containing 5% non-fat milk was performed to block the NC membranes at room temperature for 2 h after transferring the proteins onto NC membranes. Then, membranes were incubated with primary antibodies overnight at 4 ℃ followed by incubating with secondary antibodies for 1 h at room temperature. The ECL reagent was used to detect the protein bands and Image J was used to quantify the Protein bands. Details of antibodies used in this study are provided in Supplementary. Table S2.

### Quantitative real‑time (qRT)-PCR

According to the manufacturer’s instruction, total RNA was extracted from HCC cells using the RNA-Quick Purification Kit (Eesunbio, ES-RN001, China), qRT-PCR was performed using complementary DNA (cDNA) Synthesis SuperMix (Yeasen, 11141ES60, China) and SYBR Green Master Mix (Yeasen, 11184ES08, China). The 2^−ΔΔCt^ method was conducted to calculate the levels of relative gene expression. Primers employed in qPCR are shown in Supplementary. Table S3.

### RNA-sequencing

RNA-sequencing analyses were conducted to compare the DLAT knock-downed Huh7 cell samples with the control samples. Total RNA was extracted from each sample and RNA-sequencing analyses were performed on the Novaseq 6000 platform. The criteria that|log2FC| > 1 and *p* < 0.05 was identified as the differentially expressed genes (DEGs).

### Chromatin immunoprecipitation (ChIP)-qPCR

The process of ChIP was performed using the ChIP Assay Kit (Beyotime Biotechnology, P2080S, China) according to the manufacturer’s instructions. The H3K18ac antibody was used for the immunoprecipitation and qRT-PCR was performed to quantify the fold enrichment.

### Animal experiments

All animal experimental protocols were approved by the Institutional Animal Care and Use Committee of Zhejiang Cancer Hospital. All the mice were 4- 6week-old BALB/C male mice in the experiments. Briefly, 4–5 × 10^6^ Huh7 or MHCC-97 L cells suspended in 100 µL PBS were injected into the same site of the left liver lobe of nude mice to develop orthotopic liver tumors. 4–5 weeks after injection, the mice were euthanized, livers were isolated from each group of mice and the number of intrahepatic metastatic tumors excluding the primary tumor in each liver was counted to reflect the metastatic capability of the injected cell lines. To evaluate the antitumor effect of BAY-876, after 4–5 weeks of injection, the mice were divided into control and experimental groups (5 mice/group) with a similar range of bioluminescence based on in vivo imaging. Mice were dosed orally with 5 mg/kg BAY-876 once daily for 2–4 weeks.

### Statistical analysis

All data were presented as the mean ± standard error of mean (SEM). Between-group differences were compared by Student’s t-test or one way ANOVA using GraphPad Prism 8. The chi-square test was used to assess the correlation between DLAT/GLUT1 and clinicopathological features. The correlation between the IHC score and DLAT/GLUT1 was analyzed using Pearson’s correlation test. The plotting and statistical analysis of the survival curves were performed by the Kaplan-Meier method and the log-rank test, respectively. A Cox-regression model was used for multivariate analysis, and independent prognostic factors were screened. Statistical significance was defined as ^*^ p-value ≤ 0.05, ^**^ p-value ≤ 0.01, ^***^ p-value ≤ 0.001.

## Results

### High level of DLAT expression is associated with poor prognosis in HCC

**Fig. 1:**
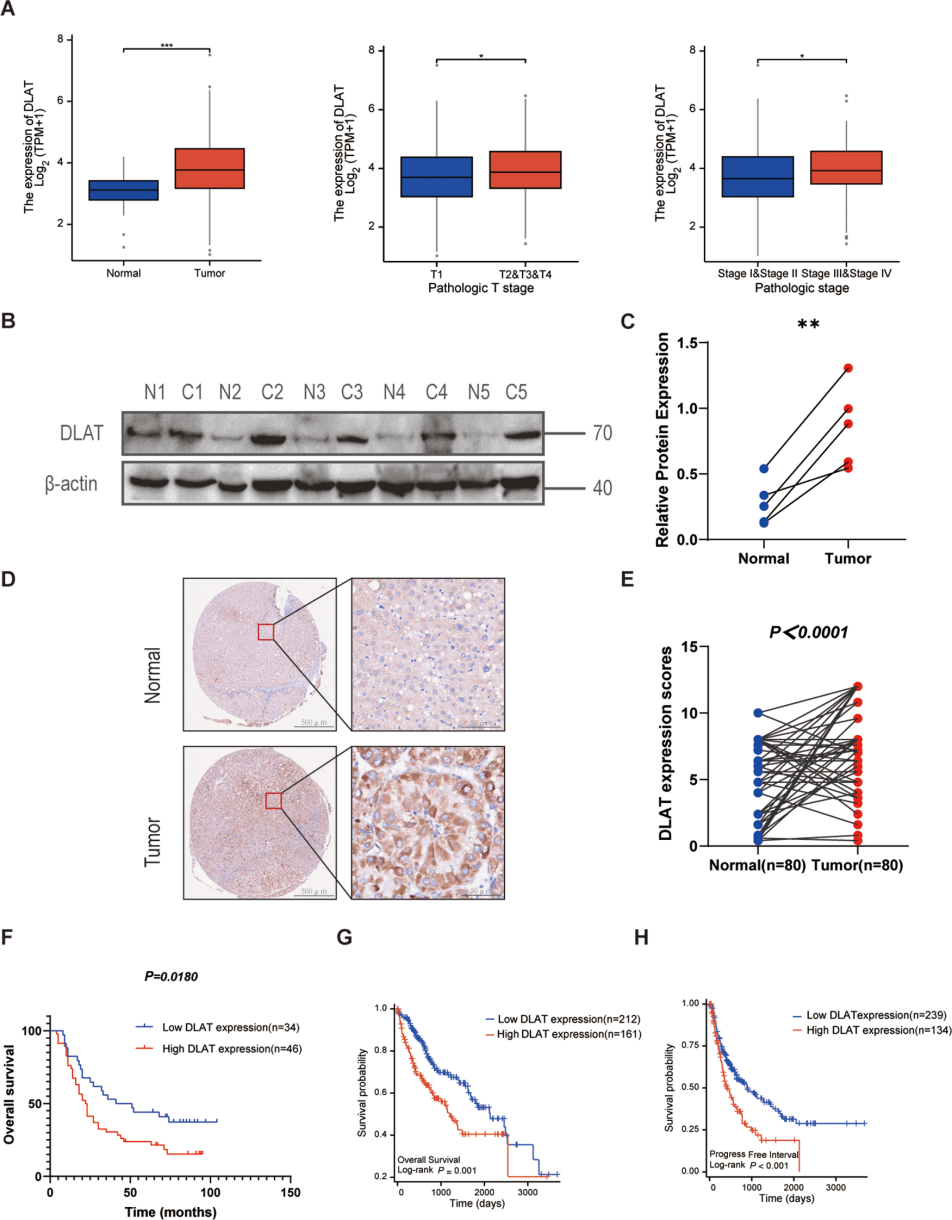
High expression of DLAT is associated with poor prognosis in HCC. **A.** Expression of the DLAT was significantly higher in HCC tissues than in adjacent normal tissues, and was elevated in tumour tissues with increasing T-stages and TNM stages of the tumour primary site. **B**,** C.** Five pairs of HCC tissues and adjacent normal tissues were subjected to western blotting analysis with the indicated antibodies. **D**,** E.** Relative DLAT expression in 80 pairs HCC(T) tissues and non-tumor (N) tissues determined by immunohistochemistry (IHC). **F.** Overall survival (OS) curve of 80 HCC patients classified by IHC scores was analyzed by Kaplan–Meier plot (*n* = 80, log-rank test). **G**,** H.** Kaplan–Meier curves of OS and progress free interval (PFS) for HCC patients with high or low DLAT expression based on TCGA database. Scale bars: 50 μm. ^*^p < 0.05 ^**^p < 0.01, ^***^p < 0.001

Previous studies have reported that elevated DLAT expression had a significantly poor prognosis in multifarious tumors(Xu et al. 2023; Li et al. 2023a; Tian et al. 2023; Yang et al. c). Consistently, we observed that DLAT expression was significantly up-regulated in HCC tissues compared to adjacent non-cancerous tissues (Fig. 1A). High DLAT expression was associated with T classification and TNM classification compared with the adjacent non-cancerous tissues by analyzing the TCGA dataset (Fig. 1A). The analysis of the correlation between DLAT expression and clinicopathologic characteristics in 80 HCC patients verified consistent trends in T classification (Table 1). We next performed the western blotting to analyze the clinical relevance of DLAT by using a cohort of 5 pairs of HCC and para-tumor (non-cancerous) tissues. The results showed that DLAT expression level was significantly higher in HCC tissues compared with para-tumor tissues (Fig. 1B, C). The analysis of IHC for 80 paraffin-embedded HCC and para-tumor tissues also showed that positive staining for DLAT proteins was more abundant in tumor tissues (Fig. 1D, E). To further investigate the relationship between DLAT and clinical characteristics, we divided the HCC patients into low DLAT expression and high DLAT expression groups based on IHC scores. Kaplan–Meier survival analysis revealed that patients with high DLAT expression presented a decreased trend in overall survival (OS) (Fig. 1F) and disease-free survival (DFS) (Fig. S1A) compared with those with low DLAT expression. Analysis of publicly available TCGA database verified consistent trends in OS and DFS (Fig. 1G, H). These results reveal that DLAT is critically involved in HCC progression and may serve as a prognostic marker.

**Table 1:** The relationship between DLAT expression and clinicopathological features in 80 HCC patients

Clinicopathologic feature	DLAT expression(*n* = 80)		*P*
	low	high	
All cases	34	46	
Ages(years)			
≤ 60	30	38	*P* = 0.5437
>60	4	8	
Gender			
Male	33	39	*P* = 0.1290
Female	1	7	
Depth of invasion			
T1	17	9	*P* = 0.0073
T2-T4	17	37	
Lymph node metastasis			
N0	33	41	*P* = 0.2333
N1/N2	1	5	
Distant metastasis			
M0	28	35	*P* = 0.5868
M1	6	11	
TNM stage			
I/II	23	21	*P* = 0.0694
III/IV	11	25	

^*^P values are from χ^2^ test

### DLAT facilitates HCC cells proliferation, motility, and migration in vitro and vivo

**Fig. 2:**
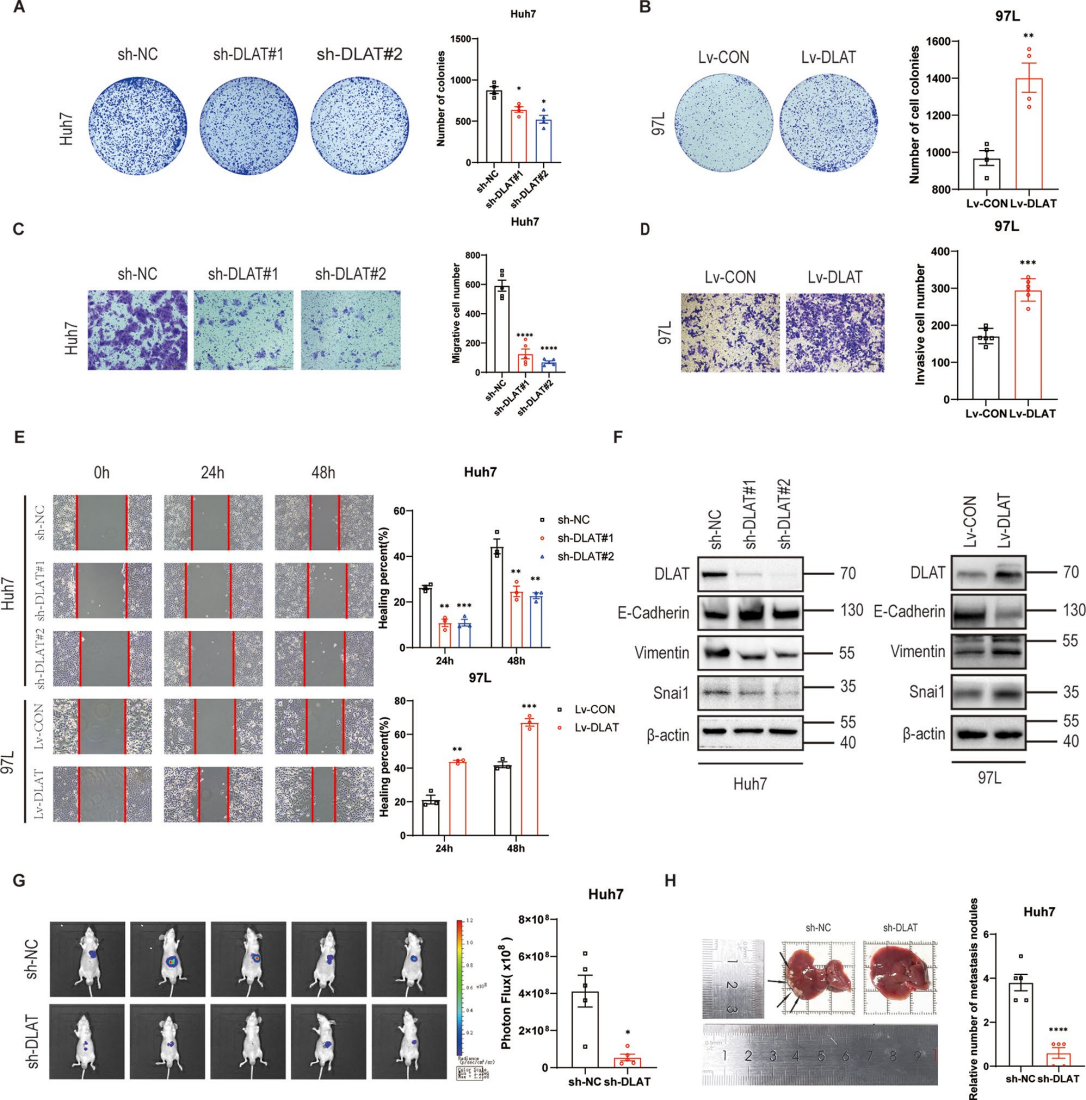
DLAT promotes HCC cell proliferation, migration, motility, and metastasis in vitro and in vivo. **A**,** B.** The proliferation of Huh7 cells and 97 L cells were conducted by colony formation. **C**,** D.** Transwell assays for Huh7 and 97 L cells indicated migration changes associated with DLAT expression. **E.** Wound healing assays for Huh7 and 97 L cells indicated motility changes linked to DLAT expression. **F.** Western blot showed the protein levels of EMT-associated makers and Snail as indicated. **G**,** H.** Typical bioluminescence images and specimens of metastasis in livers for indicated Huh7 cells. Scale bars: 200 μm. ^*^p < 0.05 ^**^p < 0.01, ^***^p < 0.001

Based on DLAT protein expression levels in five HCC cell lines (Fig.S1B), we selected the highest DLAT-expressing Huh7 cells and low metastatic MHCC-97L (97L) cells with the lowest DLAT-expression to explore the function of DLAT in the HCC progression. Stable DLAT knockdown in Huh7 cells and stable DLAT overexpression in 97 L cells were established (Fig. S1C, S1D). Huh7 cells infected with shRNA targeting DLAT (sh-DLAT#1 and sh-DLAT#2) were used to conduct a loss-of-function analysis. The result of colony formation showed that the inhibition of DLAT could impeded the proliferation of Huh7 cells (Fig. 2A). Moreover, DLAT depletion also greatly attenuated the migration (Fig. 2C) and motility potential (Fig. 2E) of Huh7 cells. Conversely, the overexpression of DLAT could promote 97 L cells proliferation (Fig. S1F) and upregulate 97 L cells colony formation (Fig. 2B). To evaluate the effect of DLAT overexpression in 97 L cells migration, Transwell migration and wound-healing assays were performed. The data indicated that DLAT overexpression significantly enhanced 97 L cells migration (Fig. 2D) and motility potential (Fig. 2E). These findings suggested that DLAT expression might be more closely associated with the metastasis of HCC than with its proliferation.

Given the critical role of epithelial-to-mesenchymal transition (EMT) in the metastasis of malignant tumors, we further asked for the relationship between DLAT and EMT in HCC by western blotting. The inhibition of DLAT in Huh7 cells increased the E-cadherin expression and reduced the vimentin expression, whereas the opposite results were observed in 97 L-DLAT-overexpression cells (Fig. 2F and Fig. S1G, H). Numerous studies have shown that transcription factors, such as Snail, Slug, and Zeb1, can regulate EMT markers(Puisieux et al. 2014; Stemmler et al. 2019). Our results of RT-qPCR revealed that only Snail mRNA level was significantly downregulated after DLAT depletion (Fig. S1I). Western blotting confirmed that Snail expression was reduced upon DLAT knockdown and increased following DLAT overexpression (Fig. 2F and Fig. S1G, H).

To further clarify the role of DLAT in the metastasis of HCC, an orthotopic liver tumor model was established in nude mice. We inject luciferase-labelled Huh7 cells with or without DLAT knockdown into the left lobe of liver in nude mice. Comparing with normal nude mice group, the luciferase signals of the liver were significantly reduced in DLAT depletion nude mice group (Fig. 2G). Less metastasis nudes in Liver were also found in DLAT depletion group compared to normal group (Fig. 2H). Taken together, our data indicate that DLAT is functionally important in regulating HCC metastasis both in vitro and vivo.

### DLAT promotes HCC cells aerobic glycolysis in vitro

**Fig. 3:**
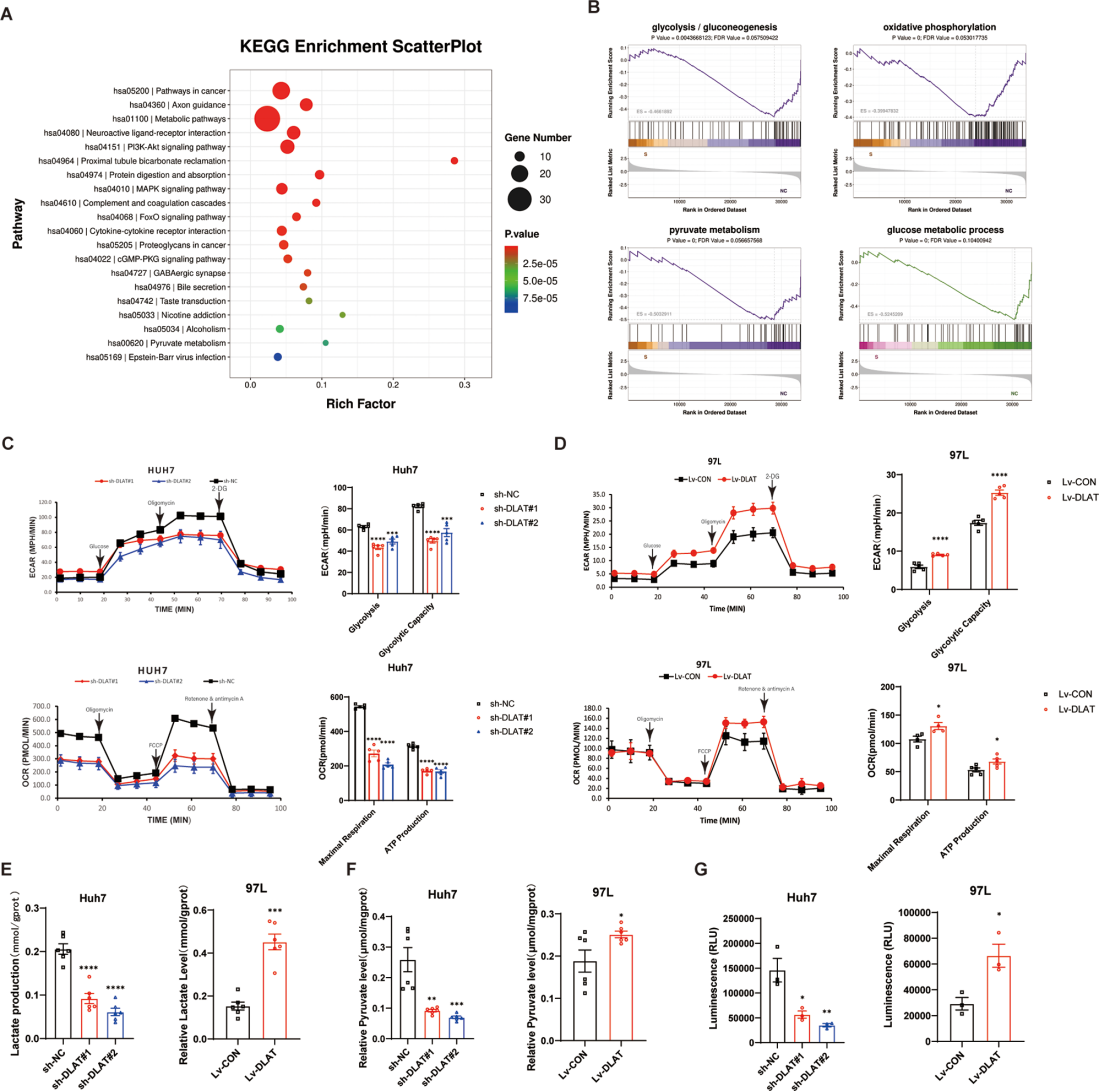
DLAT facilitates aerobic glycolysis in HCC cells. **A**,** B.** RNA-seq indicated that metabolic pathways were the top differentially expressed signaling pathways between DLAT knockdown group and the control group. Glycolysis, oxidative phosphorylation, pyruvate metabolism, and glucose metabolism were significantly downregulated in DLAT-depleted cells compared to control cells by the analysis of GSEA. **C**,** D.** Glycolysis and glycolysis capacity in Huh7 and 97 L cells with different DLAT expression levels were examined on the basis of ECAR. ATP production and maximal respiration of Huh7 and 97 L cells with different DLAT expression levels were verified using OCR. **E-G.** Relative intracellular lactate production, pyruvate levels and glucose uptake ability changes with different DLAT expression levels. ^*^p < 0.05 ^**^p < 0.01, ^***^p < 0.001

As a hub linking aerobic glycolysis and the TCA cycle, the role of DLAT in the glycolysis reprogramming of HCC remains largely unknown. Thus, we determined whether inhibition of DLAT corrects glycolysis reprogramming in HCC. KEGG pathway enrichment analysis of RNA-seq data revealed significantly metabolic pathway alterations following DLAT knockdown (Fig. 3A). Furthermore, gene set enrichment analysis (GSEA) revealed that *glycolysis/gluconeogenesis*, *oxidative phosphorylation*, *pyruvate metabolism*, and *glucose metabolic process* pathway genes were significantly down-regulated in DLAT knockdown cells compared to NC control cells (Fig. 3B). ECAR and OCR assays were performed to examine glycolytic activity and oxidative phosphorylation. As is shown in the Fig. 3C, the inhibition of DLAT weakened glycolysis and glycolysis capacity, whereas DLAT overexpression enhanced these parameters (Fig. 3D). Notably, inhibition of DLAT also impeded ATP production and maximal respiration (Fig. 3C), whereas the inverse results were observed after the overexpression of DLAT (Fig. 3D). To further validate these findings, intracellular lactate levels, intracellular pyruvate levels and glucose uptake were detected. Downregulation of DLAT led to decreased lactate production, lower pyruvate levels and reduced glucose uptake capacity (Fig. 3E-G). Conversely, increased lactate production, higher pyruvate levels and improved glucose uptake capacity (Fig. 3E-G). PDC are key rate-limiting enzymes in glycolysis and perturbation of the PDK-PDC axis can lead to a “glycolytic shift” (Stacpoole 2017). Interestingly, changes in the expression of DLAT, which is the E2 component of PDC, did not affect PDC activity (Fig. S2A, B). Therefore, these findings reveal that DLAT regulates aerobic glycolysis without affecting the PDC activity, particularly lactate production, which facilitates HCC progression.

### Glycolysis-mediated lactate promotes HCC cell metastasis in vitro

**Fig. 4:**
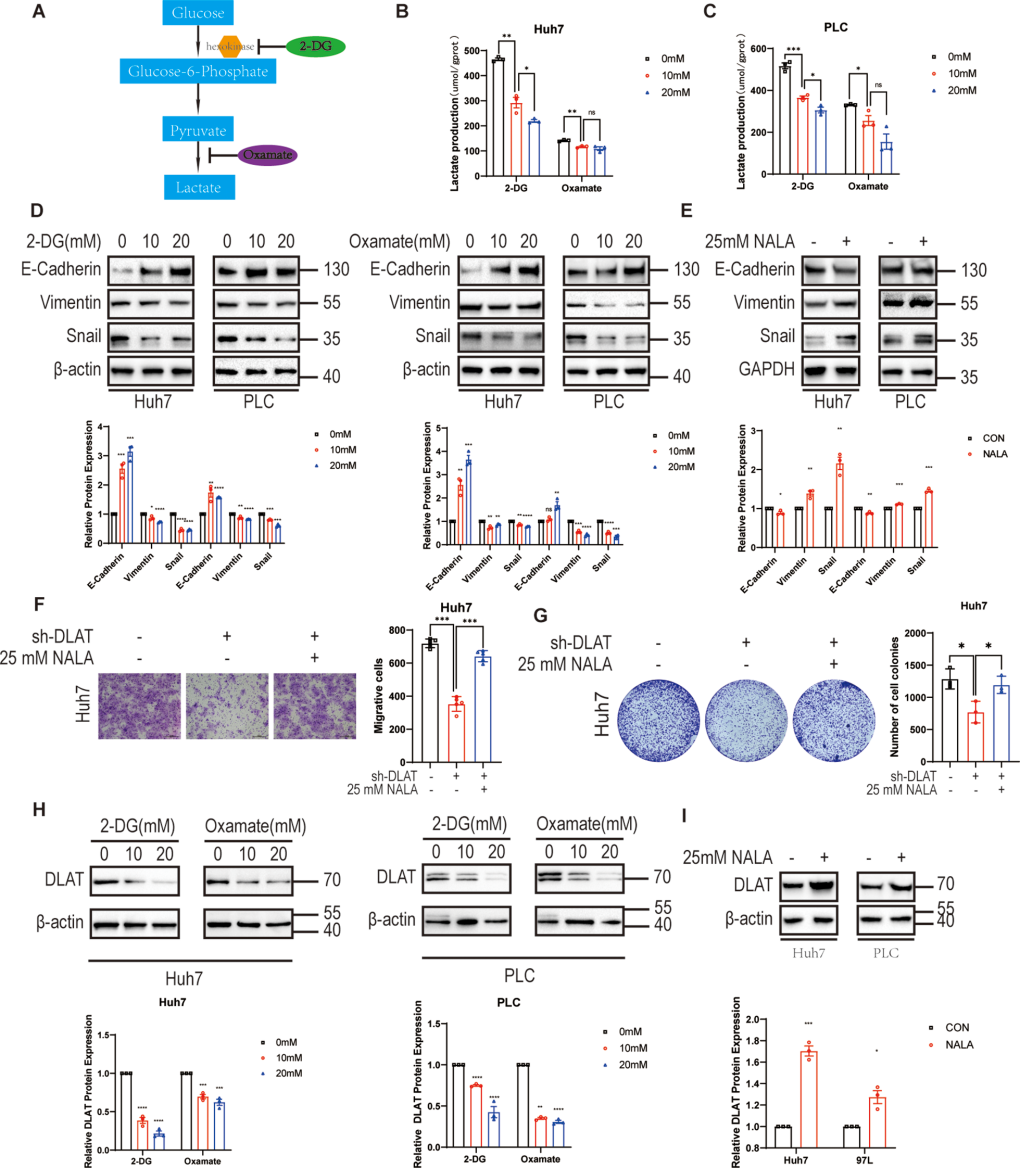
Glycolysis-mediated lactate promotes metastasis in HCC cells. **A-C**. Intracellular lactate levels in Huh7 and PLC cells were determined after 24 h of incubation with different concentrations of 2-DG or oxamate using a lactate colorimetric kit. **D**,** E.** Relative protein levels of EMT-related makers and transcription factor Snail were determined after 24 h of incubation with 2-DG, oxamate, and sodium lactate using western blotting. **F**,** G.** Transwell and colony formation assays were conducted using DLAT knockdown cells supplemented with sodium lactate. **H.** DLAT expression levels were detected in Huh7 and PLC cells cultured with different concentrations of 2-DG or oxamate for 24 h by Western blot. **I.** Western blotting determined protein levels of DLAT in Huh7 and PLC cells cultured with sodium lactate for 24 h. Scale bars, 200 μm. ^*^p < 0.05 ^**^p < 0.01, ^***^p < 0.001

To evaluate whether lactate promotes metastasis in HCC cells, intracellular lactate level in HCC cells was increased or decreased. Herein, sodium lactate was adopted to elevate lactate level, while glycolysis inhibitors (the non-metabolizable glucose analog 2-deoxy-D-glucose (2-DG) and oxamate) were used to suppress lactate production. As is shown in the Fig. 4A-C, a significant dose-dependent reduction in intracellular lactate production was observed in Huh7 and PLC cells after the treatment of 2-DG and oxamate. We first explored the relationship between lactate and EMT in HCC cells using the western blot. Increased E-cadherin protein level, decreased vimentin level and reduced Snail levels were observed in a dose-dependent manner after the treatment of 2-DG and oxamate (Fig. 4D). Conversely, sodium lactate treatment produced the opposite effect (Fig. 4E). To confirm whether the reduction in lactate resulting from DLAT knockdown has an effect on progression of HCC, lactate rescue assays were carried out. The results of Transwell and colony formation assays showed that adding back sodium lactate into DLAT depletion Huh7 cells significantly reversed cellular migration (Fig. 4F) and proliferation (Fig. 4G). The protein levels of E-cadherin, vimentin and Snail changed after lactate rescue assays (Fig. S2C). Interestingly, sodium lactate could reverse the protein level of DLAT in DLAT-knockdown cells (Fig. S2C). We further investigated the effects of lactate in the expression of DLAT. Glycolysis inhibition with 2-DG and oxamate reduced DLAT protein expression in a dramatically dose-dependent decrease (Fig. 4H). Conversely, the protein expression of DLAT were significantly upregulated after the treatment of sodium lactate (Fig. 4I). These results indicate that the changes in level of lactate due to DLAT may play a positive feedback regulation on DLAT. In a word, lactate promotes HCC progression and DLAT-mediated HCC cell metastasis is induced by lactate.

### DLAT mediates histone acetylation to regulate GLUT1 expression

**Fig. 5:**
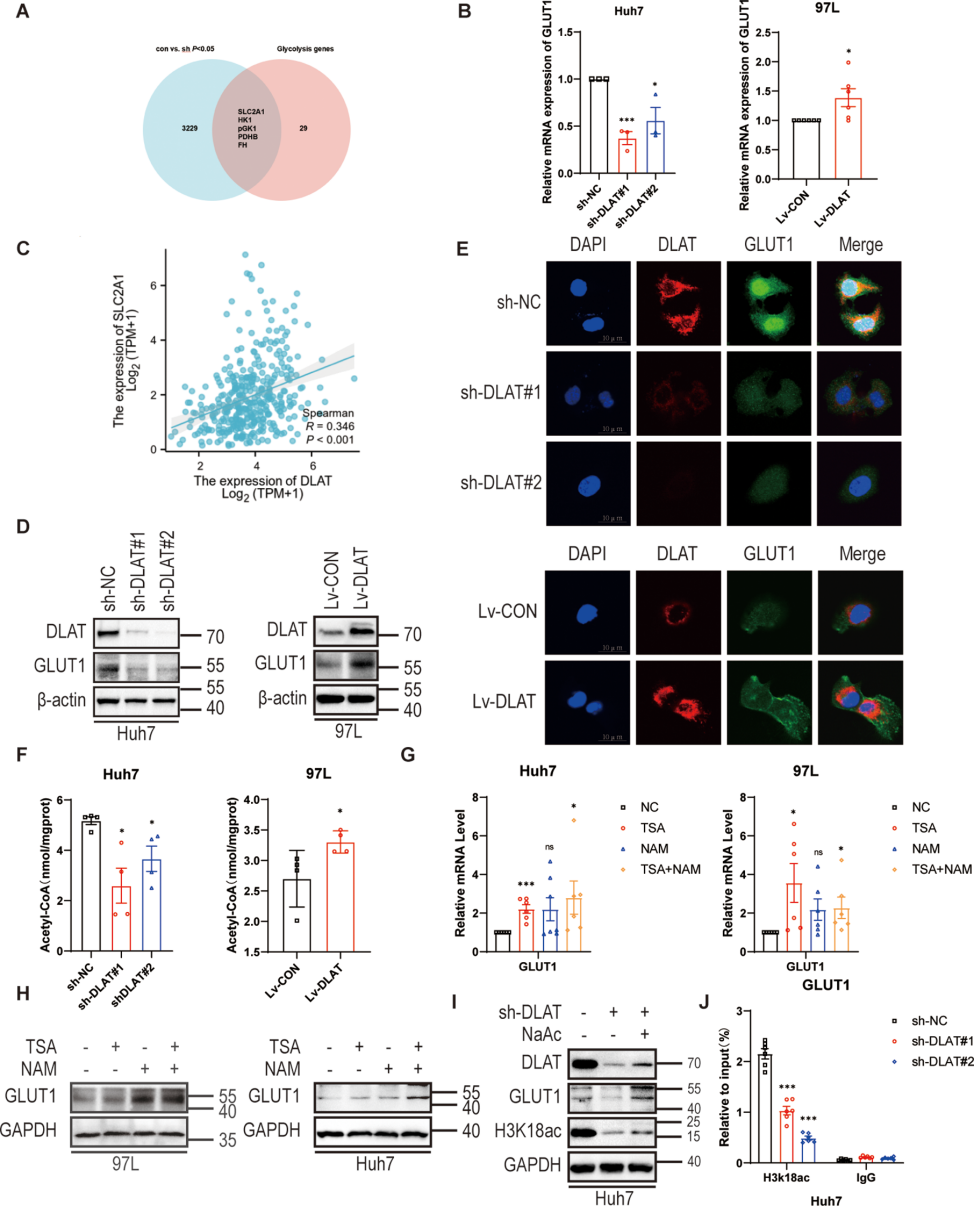
DLAT upregulates the expression of GLUT1 via histone acetylation. **(A)** RNA-seq database screened for genes related to the glycolytic pathway with differences, represented in a Wayne diagram. **(B)** Relative mRNA levels of GLUT1 were measured using RT-qPCR. **(C)** The interaction between DLAT and GLUT1 using TCGA database. **D**,** E.** Western blot and immunofluorescence showed that DLAT expression regulated GLUT1 expression in indicated cell lines. **F.** Intracellular acetyl-CoA levels were examined as indicated. **G**,** H.** RT-qPCR and western blot indicated that the GLUT1 expression was significantly upregulated when overall cellular acetylation levels increased. **I.** Western blotting indicated changes in protein levels following sodium acetate treatment. **J.** ChIP-qPCR was used to detect H3K18ac enrichment on the GLUT1 gene in Huh7 cells after DLAT depletion

To further explore the underlying mechanisms by which DLAT-mediated lactate elevation promotes HCC cell metastasis, RNA sequencing identified five key glycolysis -related genes down-regulated by DLAT knockdown (Fig. 5A). We further focused on the influences of DLAT on five candidate glycolysis-related genes through RT-qPCR. Among these, GLUT1 was significantly modulated at the mRNA level by DLAT knockdown and overexpression (Fig. 5B and Fig. S3A, B). GLUT1 was selected as a candidate gene for the next experiment. Then, Gene expression correlation analysis showed that DLAT expression positively correlated with GLUT1 based on TCGA database (Fig. 5C). Western blotting and immunofluorescence analysis further validated the regulatory role of DLAT on the expression of GLUT in Huh7 and 97 L cells (Fig. 5D, E and Fig. S3C-F).

DLAT facilitates acetyl-CoA production by transferring the acetyl group from pyruvate to coenzyme A, a process critical for histone acetylation(Peng et al. 2016; Moussaieff et al. 2015; Shi and Tu 2015). Inhibition of DLAT significantly decreased the abundance of acetyl-CoA in Huh7 cells, whereas the abundance of acetyl-CoA was enriched after the up-regulation of DLAT in 97 L cells (Fig. 5F). Furthermore, western blotting assays showed that deletion of DLAT significantly reduced the overall acetylation level in Huh7 cells, whereas the opposite result could be observed in DLAT-overexpressed 97 L cells (Fig. S3G). Previous studies have shown that DLAT acts as an acetyltransferase involved in the regulation of non-histone acetylation(Shan et al. 2014). To investigate the influence of acetylation on GLUT1, Huh7 cells and 97 L cells were treated with the deacetylase inhibitors trichostatin A (TSA) and nicotinamide (NAM), which led to increased overall acetylation in cells. Both TSA and NAM treatment significantly increased mRNA and protein levels of GLUT1 (Fig. 5G, H and Fig. S3H). Moreover. western blotting further suggested that the inhibition of DLAT reduced histone H3 acetylation at the K18(H3K18ac) and addition of sodium acetate reversed the reduction in H3K18ac (Fig. 5I and Fig. S3I). To further verify the effect of DLAT-mediated H3K18ac on GLUT1, ChIP assay was performed. The results of ChIP-qPCR showed that the depletion of DLAT dramatically reduced H3K18ac in GLUT1 gene (Fig. 5J).

### GLUT1 deficiency diminishes DLAT-mediated metastasis and aerobic glycolysis in HCC

**Fig. 6:**
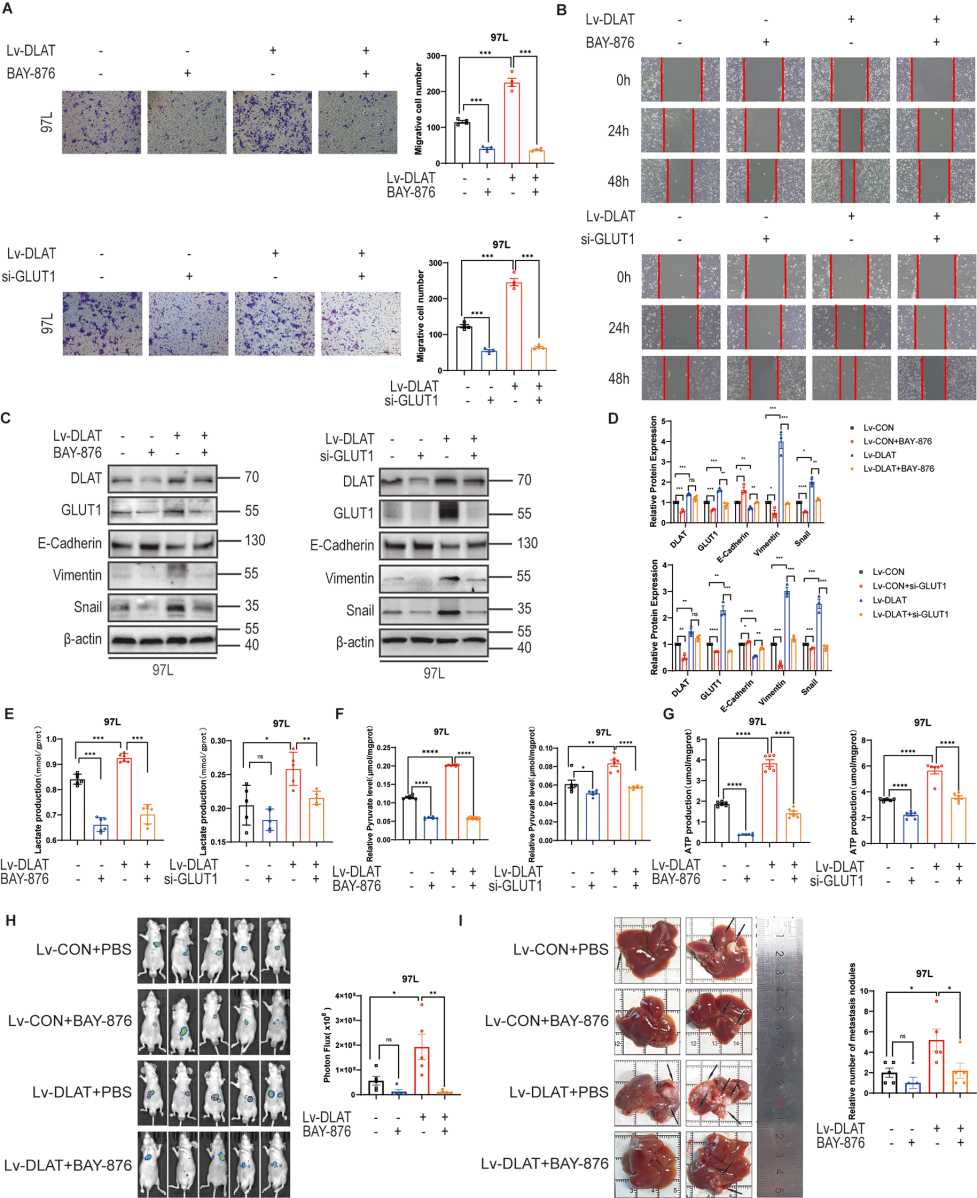
Knockdown of GLUT1 abolishes DLAT-mediated migration, motility, and glycolysis in vitro and in vivo. HCC cells infected with DLAT-overexpressing lentivirus were treated with BAY-876 or si-GLUT1. **(A)** Typical images of Transwell assays for the indicated cell lines. **(B)** Representative images of wound-healing assays for indicated cell lines. **C**,** D.** Western blotting showed changes in expression level of indicated proteins. **E-G.** Relative intracellular lactate production, pyruvate levels and ATP production were performed as indicated. **H**,** I.** Typical bioluminescence images and specimens of metastasis in livers for indicated 97 L cells. Scale bars: 200 μm. ^*^p < 0.05, ^**^p < 0.01, ^***^p < 0.001

To determine the role of GLUT1 in DLAT-mediated metastasis and aerobic glycolysis, the expression of GLUT1 in DLAT-overexpression cells was depleted using GLUT1 inhibitor(BAY-876)and siRNA. The results of Transwell assays suggested that the deficiency of GLUT1 dramatically abrogated DLAT-related cell migration (Fig. 6A). Inhibition of GLUT1 could also abolish the motility potential of DLAT-related cells (Fig. 6B). Moreover, relative protein levels of DLAT-mediated EMT dramatically were reversed by BAY-876 and siGLUT1 (Fig. 6C, D). We then explored the effect of GLUT1 on aerobic glycolysis by the analysis of lactate, pyruvate, and ATP assays when DLAT is overexpressed. Both BAY-876 and siGLUT1 significantly mitigated the increase in lactate (Fig. 6E), pyruvate (Fig. 6F), and ATP (Fig. 6G) levels induced by the up-regulation of DLAT. To further explore the effect of the DLAT/GLUT1 pathway on HCC metastasis in vivo, we constructed orthotopic HCC models in nude mice. The liver metastasis was significantly enhanced with DLAT stimulation, whereas the progression was significantly diminished by GLUT1 inhibition (Fig. 6H and I).

These results reveal that the aggressive characteristics of HCC caused by DLAT up-regulation are diminished by the inhibition of GLUT1, which suggests GLUT1 is an indispensable key factor for DLAT to regulate aerobic glycolysis and promote tumor metastasis.

### DLAT expression correlates with GLUT1 in HCC

**Fig. 7:**
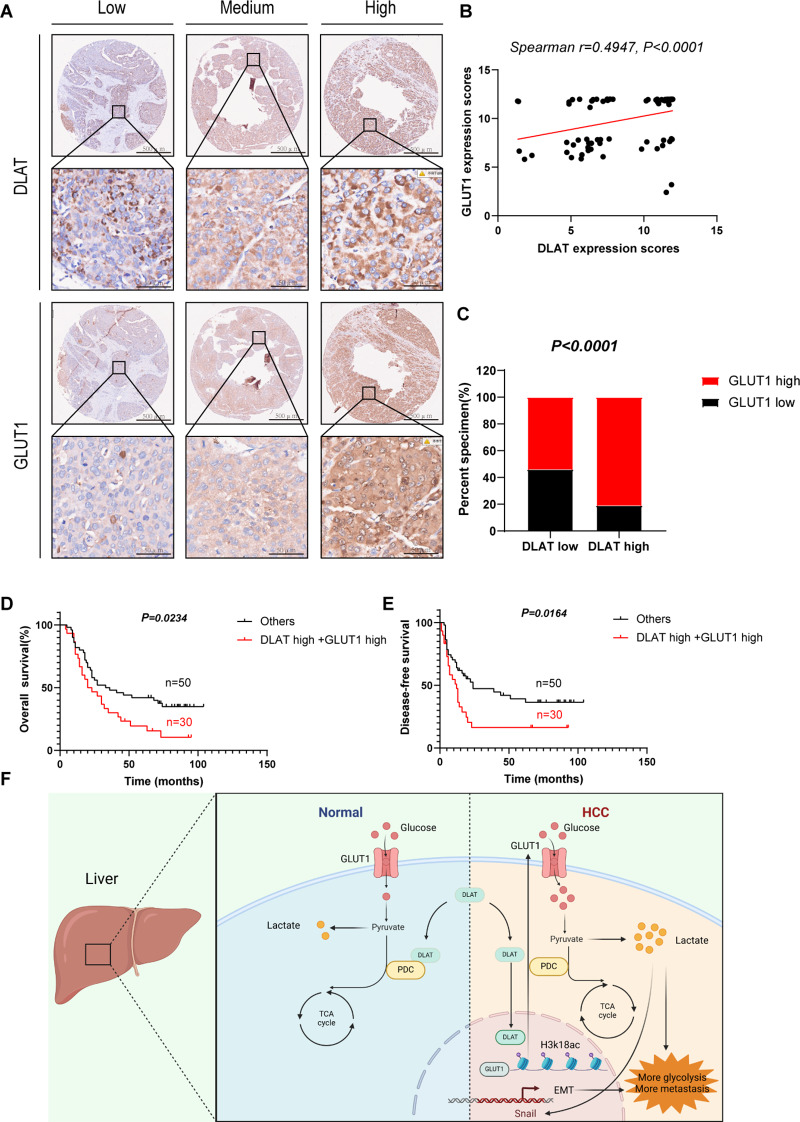
Elevated DLAT and GLUT1 accumulation is positively associated with poor prognosis in patients with HCC. **(A)** Representative images of IHC staining for DLAT and GLUT1 in 80 HCC tissues. **(B)** Correlation analysis indicated that DLAT was positively correlated with GLUT1, spearman correlation analysis was used. (*n* = 80) **(C)** Correlation between DLAT and GLUT1 were examined using Fisher’s exact test, respectively. (*n* = 80) **D**,** E.** Kaplan-Meier survival curves of overall survival (OS) and disease-free survival (DFS) in HCC patients with high DLAT combined with high GLUT1. (*n* = 80). **F.** Schematic illustration depicting how DLAT activates EMT to promote HCC metastasis by regulating GLUT1-mediated glycolytic reprogramming, created using Biorender. (www.biorender.com). Scale bars: 50 μm

Given the regulatory relationship between DLAT and GLUT1, we further analyzed their abundance in HCC tissues. As is shown in the Fig. 7A, the results of IHC showed that DLAT expression was positively correlated with that of GLUT1 in tumor tissues. We also confirmed that high DLAT expression was positively correlated with high GLUT1 expression (Fig. 7B, C). Moreover, HCC patients with high DLAT and GLUT1 co-expression presented with comparatively worse prognoses (Fig. 7D, E). Taken together, compared with DLAT alone, the combination of two factors achieves a higher prognostic value.

## Discussion

HCC, which has high morbidity and mortality rates, is the third leading cause of cancer death globally (Singal et al. 2023). Despite the fact that the treatment of HCC has progressed considerably over the past decade, the majority of HCC patients have a poor prognosis with high rate of recurrence, metastasis, and the resistance to systemic therapy (Yang et al. 2021; Yu et al. 2022). This underscores the critical need to identify reliable biomarkers for improving diagnosis and therapeutic intervention of HCC. A typical hallmark of metabolic reprogramming is the aberrant activation of glycolysis and has been reported to promote HCC tumorigenesis and progression (Du et al. 2022). Thus, understanding the molecular links between aerobic glycolysis and aggressive phenotypes in HCC has great clinical value.

DLAT, the E2 subunit of the mitochondrial PDC, is a key cuproptosis-related signature (CRS) gene that has been involved in multiple tumor initiation and progression (Chen et al. 2022; Li et al. 2023a, b). Moreover, DLAT has also been shown to be associated with biliary cholangitis, embryonic development, metabolism and cell stemness (Terziroli Beretta-Piccoli et al. 2018; Tsvetkov et al. 2022; Goguet-Rubio et al. 2016). In this study, we demonstrated that up-regulated DLAT was associated with malignancy and poor prognosis in HCC by utilizing public databases and verified this finding with clinical tissue samples collected. These findings align with previous studies (Li et al. b). We further confirmed that ectopic expression of DLAT dramatically increased the metastasis and aerobic glycolysis of HCC cells in vitro and vivo. Its depletion led to reduced levels of pyruvate, lactate production, and glucose uptake in HCC, mirroring observations in lung cancer (Chen et al. 2022). Our RNA sequencing data confirmed that DLAT enhanced aerobic glycolysis and acted as a metabolic reprogramming molecule involved in tumor metastasis. Hypoxia, a hallmark of solid tumors, including HCC, is known to influence glucose metabolism and increase lactate production (Chen et al. 2023). While this study focused on aerobic glycolysis, whether it is possible to lead to remodeling of glucose metabolism and increased lactate production by targeting DLAT in the hypoxic microenvironment warrants further investigation. Understanding the relationship between DLAT and anaerobic glycolysis could provide new insights into its role in tumor metabolism.

Lactate, a compound generated during glycolysis, is generally recognized as a source of energy and a metabolic by-product (Zhang et al. 2019). However, a growing amount of research suggests that lactate contributes to cancer metastasis by inducing the EMT process, facilitating migration, improving angiogenesis and promoting immune evasion(Chen et al. 2017). In the current study, we found that decreases and increases in lactate significantly altered EMT-related markers and the transcription factor Snail. Notably, lactate supplementation reversed the effects of DLAT depletion on HCC cells migration and EMT. Moreover, we noticed that decreases and increases in lactate can also affect DLAT expression in a positive feedback way. However, the specific molecular mechanism of this positive feedback regulation needs to be explored in the future. Collectively, these results can be view as evidence that DLAT-mediated HCC cell metastasis is induced by lactate and forms positive feedback with DLAT.

We screened molecules that played a crucial role in our study by investigating the several expressions of key enzymes of glycolysis and glucose transporter proteins in the glycolytic pathway. Based on TCGA data, the RNA sequencing data, and RT-qPCR outcomes, GLUT1 was selected as a candidate downstream gene of DLAT. GLUT1, a key glucose transporter overexpressed in multiple cancers, facilitates glucose uptake and contributes to tumor cell growth, metastasis, and other malignant behaviors (Amann et al. 2009; Pliszka and Szablewski 2021; Martinez-Outschoorn et al. 2017). Epigenetic modifications can affect GLUT1 expression, particularly the regulation of enhancer activity (Yu et al. 2019; Yao et al. 2023). While DLAT has been implicated in the regulation of non-histone acetylation(Shan et al. 2014), its role in histone acetylation remains unexplored at the present. Our findings suggested that DLAT promoted the enhancer activity of GLUT1 by H3K18ac, thereby facilitating GLUT1 expression in HCC. However, whether this regulation process is a direct binding of DLAT as an acetyltransferase or an indirect action of acetyl CoA needs to be further confirmed in future studies. BAY-876, a selective GLUT1 inhibitor, shows high affinity and selectivity for GLUT1. Although studies have reported antitumor activity of BAY-876 in breast and ovarian cancer (Wu et al. 2020; Ma et al. 2018), its role of antitumor in HCC remains unknown. We found both BAY-876 and siGLUT1 could abolish the DLAT-mediated EMT and metastasis, as well as glycolysis in HCC cells. GLUT1 inhibition could also diminish the progression of liver metastasis caused by DLAT stimulation in vivo. Finally, our correlation analyses confirmed that the combination of DLAT and GLUT1 could be utilized to predict prognosis for HCC patients and would be of high clinical importance in the future.

## Conclusion

In conclusion, our studies indicated that DLAT and GLUT1 are promising and valuable targets for the treatment of HCC. By establishing DLAT as a critical regulator of glycolysis and metastasis via the DLAT/GLUT1 axis, our findings highlight its pivotal role in metabolic reprogramming. Targeting DLAT/GLUT1 provides promising diagnostic and therapeutic strategies for HCC.

## Electronic supplementary material

Below is the link to the electronic supplementary material.

Supplementary Material 1

Supplementary Material 2

## Data Availability

No datasets were generated or analysed during the current study.

## Abbreviations

DLAT: Dihydrolipoyl transacetylase
HCC: Hepatocellular carcinoma
GLUT1: Glucose transporter 1
PDC: pyruvate dehydrogenase complex
OS: Overall survival
DFS: Disease-free survival
EMT: Epithelial-to-mesenchymal transition
2-DG: 2-deoxy-D-glucose
TSA: Trichostatin A
NAM: Nicotinamide
CRS: Cuproptosis-related signature
