# Supplementary material for: DLAT activates EMT to promote HCC metastasis by regulating GLUT1-mediated aerobic glycolysis
Source: Mol Med. 2025 Feb 20;31:71. doi: 10.1186/s10020-025-01125-5 (PMC11844032; doi:10.1186/s10020-025-01125-5)
Supplement: Supplementary file 1 — Supplementary Material 1 [file 10020_2025_1125_MOESM1_ESM.pdf]

Supplementary Material 1

DLAT activates EMT to promote HCC metastasis by regulating GLUT1-mediated glycolytic reprogramming

FigureS1.

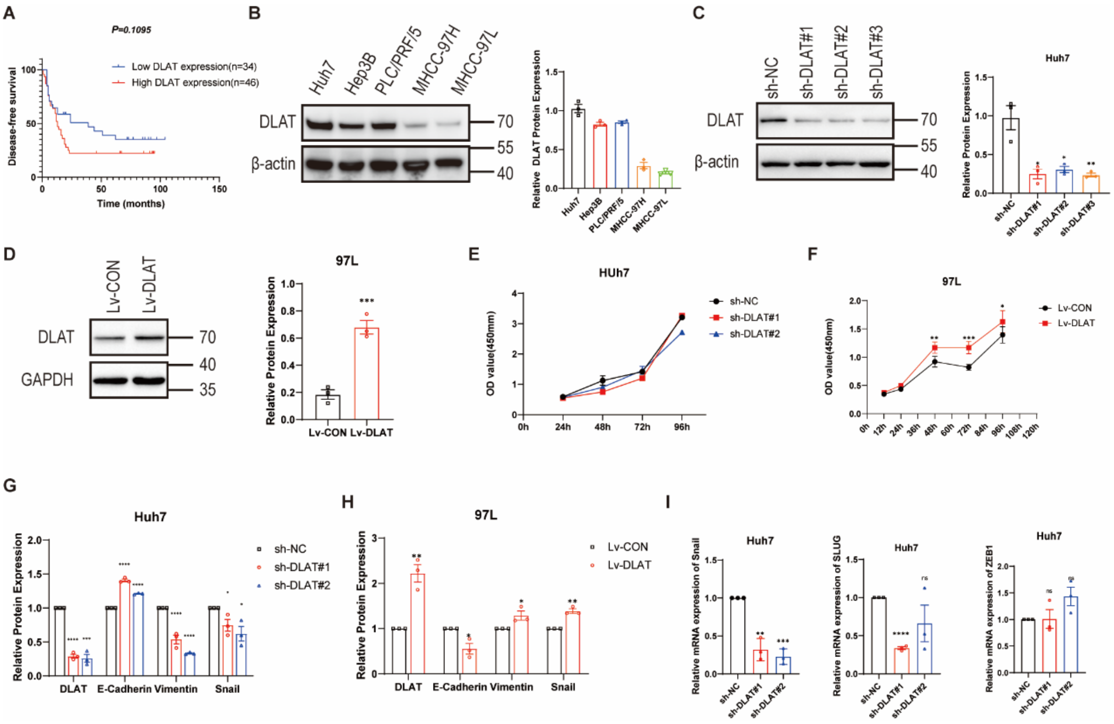

A. Disease-free survival (DFS) curve of 80 HCC patients classified by IHC scores was analyzed by Kaplan–Meier plot (n = 80, log-rank test). **B.** Relative protein level of DLAT in five HCC cell lines. **C, D.** Relative protein level of DLAT after knockdown and overexpression in Huh7 cells and 97L cells by western blotting analysis. **E, F.** The proliferation of Huh7 cells downregulating DLAT and 97L upregulating DLAT were conducted by CCK8 assays compared with the control group. **G, H.** Relative protein expression levels of DLAT, e-cadherin, vimentin, and snail. **I.** Relative mRNA expression levels of snail, slug, and zeb1 were analyzed by RT-qPCR.

Figure.S2

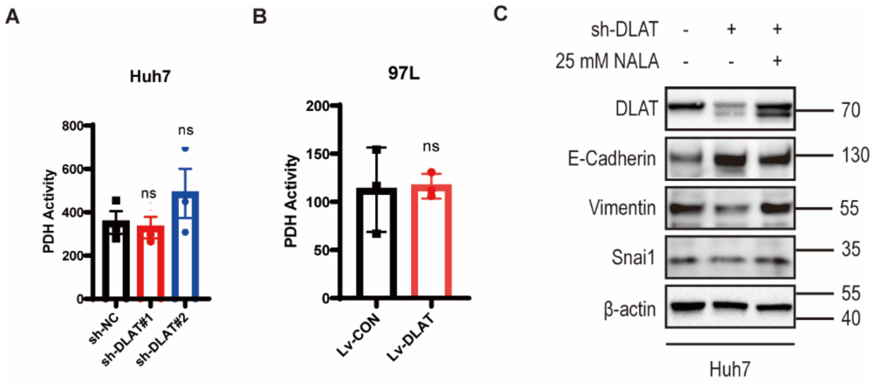

A, B. PDH activity was analyzed after DLAT knockdown or overexpression using PDH activity assay kit. **C.** DLAT, E-cadherin, Vimentin, and Snai1 protein levels were determined using western blot when sodium lactate was adopted to DLAT-knockdown cells for 24h.

Figure.S3

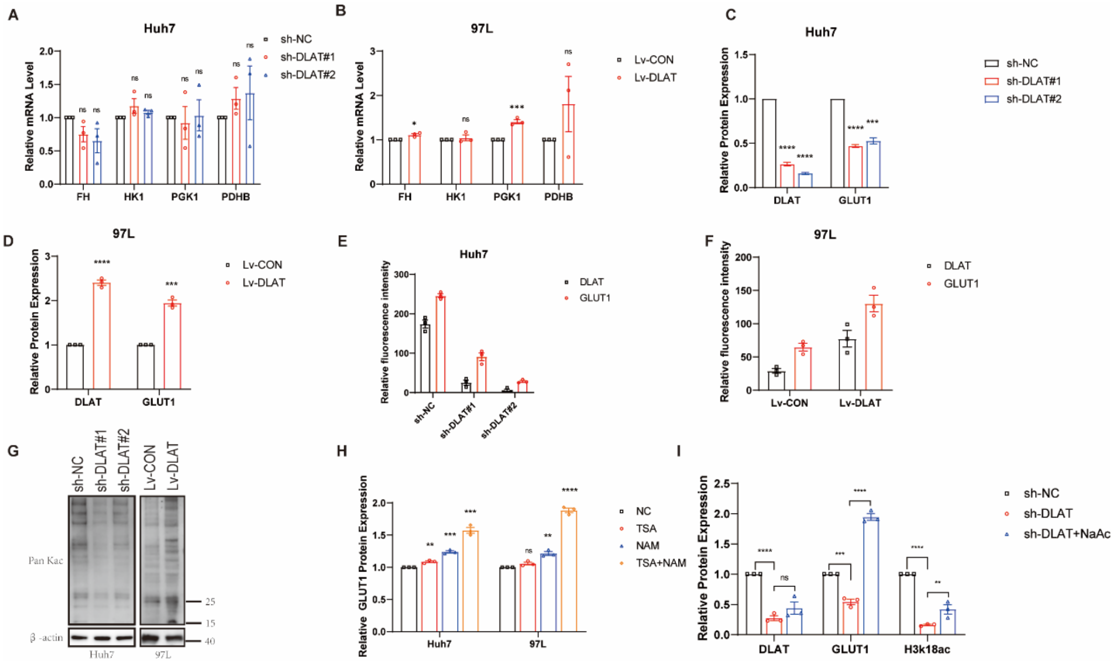

A, B. Relative mRNA levels of FH, HK1, PGK1 and PDHB were analyzed by RT-qPCR. **C, D.** Relative protein expression analysis by western blot. **E, F.** Relative fluorescence intensity of DLAT and GLUT1 were examined by Immunofluorescence. **G.** the overall acetylation level in Huh7 and 97L cells were detected by western blot. **H, I.** Relative protein expression analysis by western blot.
