# Supplementary material for: DLAT activates EMT to promote HCC metastasis by regulating GLUT1-mediated aerobic glycolysis
Source: Mol Med. 2025 Feb 20;31:71. doi: 10.1186/s10020-025-01125-5 (PMC11844032; doi:10.1186/s10020-025-01125-5)
Supplement: Supplementary file 2 — Supplementary Material 2 [file 10020_2025_1125_MOESM2_ESM.pdf]

Supplementary Material 2

DLAT activates EMT to promote HCC metastasis by regulating GLUT1-mediated glycolytic reprogramming

Supplementary. Table S1. The sequences of shRNA and siRNA used for gene silencing

RNAs	Target RNA Sequence
sh-DLAT#1	GGTGTTTGTTAGCCCTCTTGC
sh-DLAT#2	GCTTCAGCTTTGGCATGTTTA
sh-DLAT#3	GGAAACCATTGCTAATGATGT
si-GLUT1#1	GCATGTGCTTCCAGTATGT
si-GLUT1#2	CAAAGTTCCTGAGACTAAA
si-GLUT1#3	TGAGCATCGTGGCCATCTT
si-NC	ACGUGACUCGUUCGGAGAATT

Supplementary. Table S2. The information of antibodies used in this study.

**Antibody**	**Source**	**Identifier**
Mouse anti-DLAT	Abcam	ab110333
Rabbit anti-DLAT	Proteintech	13426-1-AP
Mouse anti-GLUT1	Abcam	ab115730
Rabbit anti-GLUT1	Proteintech	21829-1-AP
Rabbit anti-SNAI1	Proteintech	13099-1-AP
Rabbit anti-E-cadherin	Proteintech	20874-1-AP
Rabbit anti-Vimentin	Proteintech	10366-1-AP
Rabbit anti-Histone H3(acetyl K18)	Abcam	ab177870
Mouse anti-β-actin	Proteintech	66009-1-Ig
Mouse anti-GAPDH	Proteintech	60004-1-Ig
Rabbit anti-Acetylated-Lysine	Cell Signaling Technology	#9441

Supplementary. Table S3. Primers for RT-qPCR

Gene names	Sequences
DLAT	F-GCAGGACTCATCACACCTATTGT
	R-GTAGTTTACCCTCTCTTGCTTTGG
Snail	F-TGCCCTCAAGATGCACATCCGA
	R-GGGACAGGAGAAGGGCTTCTC
ZEB1	F-GGCATACACCTACTCAACTACGG
	R-TGGGCGGTGTAGAATCAGAGTC
SLUG	F-TGGTCAAGAAACATTTCAACGCC
	R-GGTGAGGATCTCTGGTTTTGGTA
GLUT1	F-CTGGCATCAACGCTGTCTTC
	R-GCCTATGAGGTGCAGGGTC
HK2	F-TGGAACTGGTGGAAGGAGAAGAG
	R-GTTGCAGGATGGCTCGGACTTG
PKM2	F-CTATCCTCTGGAGGCTGTGC
	R-GGGGTCGCTGGTAATG
FH	F- TGGGAATCCAGGCCAATAC
	R- GCTGCCTTGTCATACCCTAT
PGK1	F- TGGACGTTAAAGGGAAGCGG
	R- GCTCATAAGGACTACCGACTTGG
HK1	F- GTCCATTCCTGATGGCTCTG
	R- TCTCATGATTCACTTGCACCC
PDHB	F- AAGAGGCGCTTTCACTGGACA
	R- ACTAACCTTGTATGCCCCATCA
β-actin	F-CATGTACGTTGCTATCCAGGC
	R-CTCCTTAATGTCACGCACGAT
